# Supplementary material for: Pyruvate Dehydrogenase Kinase 1 inhibition mediated oxidative phosphorylation enhancement in cartilage promotes osteoarthritis progression
Source: BMC Musculoskelet Disord. 2023 Jul 20;24:597. doi: 10.1186/s12891-023-06585-6 (PMC10357736; doi:10.1186/s12891-023-06585-6)
Supplement: Supplementary file 1 — Additional file 1. [file 12891_2023_6585_MOESM1_ESM.zip › MF.Gsea.1653627316242/GOMF_ORGANIC_ANION_TRANSMEMBRANE_TRANSPORTER_ACTIVITY.html]

Details for gene set GOMF\_ORGANIC\_ANION\_TRANSMEMBRANE\_TRANSPORTER\_ACTIVITY[GSEA]

|  || Dataset | 55457\_collapsed\_to\_symbols.cls.cls #OA\_versus\_normal.cls.cls #OA\_versus\_normal\_repos |
| Phenotype | cls.cls#OA\_versus\_normal\_repos |
| Upregulated in class | normal |
| GeneSet | GOMF\_ORGANIC\_ANION\_TRANSMEMBRANE\_TRANSPORTER\_ACTIVITY |
| Enrichment Score (ES) | -0.34505177 |
| Normalized Enrichment Score (NES) | -1.5984364 |
| Nominal p-value | 0.003003003 |
| FDR q-value | 0.1559872 |
| FWER p-Value | 0.996 |
Table: GSEA Results Summary

  

Fig 1: Enrichment plot: GOMF\_ORGANIC\_ANION\_TRANSMEMBRANE\_TRANSPORTER\_ACTIVITY      
 Profile of the Running ES Score & Positions of GeneSet Members on the Rank Ordered List

  

| SYMBOL | TITLE | RANK IN GENE LIST | RANK METRIC SCORE | RUNNING ES | CORE ENRICHMENT || 1 | MPC2 | mitochondrial pyruvate carrier 2 [Source:HGNC Symbol;Acc:HGNC:24515] | 140 | 0.870 | 0.0107 | No |
| 2 | SLC25A31 | solute carrier family 25 member 31 [Source:HGNC Symbol;Acc:HGNC:25319] | 395 | 0.708 | 0.0084 | No |
| 3 | SLC2A10 | solute carrier family 2 member 10 [Source:HGNC Symbol;Acc:HGNC:13444] | 874 | 0.550 | -0.0154 | No |
| 4 | SLC27A6 | solute carrier family 27 member 6 [Source:HGNC Symbol;Acc:HGNC:11000] | 1182 | 0.480 | -0.0275 | No |
| 5 | SLC1A4 | solute carrier family 1 member 4 [Source:HGNC Symbol;Acc:HGNC:10942] | 1372 | 0.447 | -0.0312 | No |
| 6 | SLC25A11 | solute carrier family 25 member 11 [Source:HGNC Symbol;Acc:HGNC:10981] | 1448 | 0.436 | -0.0262 | No |
| 7 | SLC35A1 | solute carrier family 35 member A1 [Source:HGNC Symbol;Acc:HGNC:11021] | 1779 | 0.387 | -0.0424 | No |
| 8 | SLCO1A2 | solute carrier organic anion transporter family member 1A2 [Source:HGNC Symbol;Acc:HGNC:10956] | 1853 | 0.379 | -0.0387 | No |
| 9 | SLCO1B1 | solute carrier organic anion transporter family member 1B1 [Source:HGNC Symbol;Acc:HGNC:10959] | 2051 | 0.356 | -0.0453 | No |
| 10 | SLC35B1 | solute carrier family 35 member B1 [Source:HGNC Symbol;Acc:HGNC:20798] | 2213 | 0.337 | -0.0495 | No |
| 11 | SLC25A30 | solute carrier family 25 member 30 [Source:HGNC Symbol;Acc:HGNC:27371] | 2229 | 0.336 | -0.0423 | No |
| 12 | SLC29A1 | solute carrier family 29 member 1 (Augustine blood group) [Source:HGNC Symbol;Acc:HGNC:11003] | 2603 | 0.290 | -0.0643 | No |
| 13 | SLC5A12 | solute carrier family 5 member 12 [Source:HGNC Symbol;Acc:HGNC:28750] | 2652 | 0.285 | -0.0610 | No |
| 14 | SLC3A1 | solute carrier family 3 member 1 [Source:HGNC Symbol;Acc:HGNC:11025] | 2706 | 0.279 | -0.0582 | No |
| 15 | SLC38A6 | solute carrier family 38 member 6 [Source:HGNC Symbol;Acc:HGNC:19863] | 3060 | 0.244 | -0.0798 | No |
| 16 | SFXN3 | sideroflexin 3 [Source:HGNC Symbol;Acc:HGNC:16087] | 3087 | 0.241 | -0.0758 | No |
| 17 | SLCO5A1 | solute carrier organic anion transporter family member 5A1 [Source:HGNC Symbol;Acc:HGNC:19046] | 3132 | 0.237 | -0.0734 | No |
| 18 | SLC2A2 | solute carrier family 2 member 2 [Source:HGNC Symbol;Acc:HGNC:11006] | 3168 | 0.234 | -0.0703 | No |
| 19 | SLC25A21 | solute carrier family 25 member 21 [Source:HGNC Symbol;Acc:HGNC:14411] | 3218 | 0.228 | -0.0685 | No |
| 20 | SLC4A1 | solute carrier family 4 member 1 (Diego blood group) [Source:HGNC Symbol;Acc:HGNC:11027] | 3233 | 0.227 | -0.0639 | No |
| 21 | SLC33A1 | solute carrier family 33 member 1 [Source:HGNC Symbol;Acc:HGNC:95] | 3320 | 0.219 | -0.0652 | No |
| 22 | CTNS | "cystinosin, lysosomal cystine transporter [Source:HGNC Symbol;Acc:HGNC:2518]" | 3337 | 0.218 | -0.0610 | No |
| 23 | ABCC4 | ATP binding cassette subfamily C member 4 [Source:HGNC Symbol;Acc:HGNC:55] | 3344 | 0.217 | -0.0561 | No |
| 24 | SLCO1C1 | solute carrier organic anion transporter family member 1C1 [Source:HGNC Symbol;Acc:HGNC:13819] | 3566 | 0.197 | -0.0685 | No |
| 25 | SLC6A12 | solute carrier family 6 member 12 [Source:HGNC Symbol;Acc:HGNC:11045] | 4093 | 0.147 | -0.1061 | No |
| 26 | SLC9A3R1 | SLC9A3 regulator 1 [Source:HGNC Symbol;Acc:HGNC:11075] | 4111 | 0.145 | -0.1038 | No |
| 27 | SLC22A3 | solute carrier family 22 member 3 [Source:HGNC Symbol;Acc:HGNC:10967] | 4191 | 0.139 | -0.1065 | No |
| 28 | SLC25A6 | solute carrier family 25 member 6 [Source:HGNC Symbol;Acc:HGNC:10992] | 4211 | 0.136 | -0.1046 | No |
| 29 | SLCO2B1 | solute carrier organic anion transporter family member 2B1 [Source:HGNC Symbol;Acc:HGNC:10962] | 4311 | 0.128 | -0.1092 | No |
| 30 | MFSD10 | major facilitator superfamily domain containing 10 [Source:HGNC Symbol;Acc:HGNC:16894] | 4376 | 0.123 | -0.1112 | No |
| 31 | SLC22A2 | solute carrier family 22 member 2 [Source:HGNC Symbol;Acc:HGNC:10966] | 4435 | 0.118 | -0.1128 | No |
| 32 | SLC2A6 | solute carrier family 2 member 6 [Source:HGNC Symbol;Acc:HGNC:11011] | 4449 | 0.117 | -0.1109 | No |
| 33 | SLC26A4 | solute carrier family 26 member 4 [Source:HGNC Symbol;Acc:HGNC:8818] | 4552 | 0.109 | -0.1162 | No |
| 34 | SLC6A14 | solute carrier family 6 member 14 [Source:HGNC Symbol;Acc:HGNC:11047] | 4634 | 0.101 | -0.1200 | No |
| 35 | SLCO4C1 | solute carrier organic anion transporter family member 4C1 [Source:HGNC Symbol;Acc:HGNC:23612] | 4714 | 0.095 | -0.1238 | No |
| 36 | SLC4A4 | solute carrier family 4 member 4 [Source:HGNC Symbol;Acc:HGNC:11030] | 5027 | 0.071 | -0.1465 | No |
| 37 | SLC25A4 | solute carrier family 25 member 4 [Source:HGNC Symbol;Acc:HGNC:10990] | 5073 | 0.067 | -0.1484 | No |
| 38 | ABCC2 | ATP binding cassette subfamily C member 2 [Source:HGNC Symbol;Acc:HGNC:53] | 5206 | 0.056 | -0.1573 | No |
| 39 | MPC1 | mitochondrial pyruvate carrier 1 [Source:HGNC Symbol;Acc:HGNC:21606] | 5313 | 0.046 | -0.1645 | No |
| 40 | SLC25A14 | solute carrier family 25 member 14 [Source:HGNC Symbol;Acc:HGNC:10984] | 5333 | 0.045 | -0.1648 | No |
| 41 | SLC25A12 | solute carrier family 25 member 12 [Source:HGNC Symbol;Acc:HGNC:10982] | 5506 | 0.032 | -0.1775 | No |
| 42 | PDPN | podoplanin [Source:HGNC Symbol;Acc:HGNC:29602] | 5594 | 0.024 | -0.1837 | No |
| 43 | SLC35A3 | solute carrier family 35 member A3 [Source:HGNC Symbol;Acc:HGNC:11023] | 5802 | 0.008 | -0.1997 | No |
| 44 | SLC38A7 | solute carrier family 38 member 7 [Source:HGNC Symbol;Acc:HGNC:25582] | 5916 | 0.001 | -0.2086 | No |
| 45 | SLC16A1 | solute carrier family 16 member 1 [Source:HGNC Symbol;Acc:HGNC:10922] | 6043 | -0.008 | -0.2182 | No |
| 46 | SLC25A15 | solute carrier family 25 member 15 [Source:HGNC Symbol;Acc:HGNC:10985] | 6086 | -0.011 | -0.2213 | No |
| 47 | SLC2A8 | solute carrier family 2 member 8 [Source:HGNC Symbol;Acc:HGNC:13812] | 6137 | -0.015 | -0.2248 | No |
| 48 | SLC16A8 | solute carrier family 16 member 8 [Source:HGNC Symbol;Acc:HGNC:16270] | 6248 | -0.024 | -0.2328 | No |
| 49 | SLC39A8 | solute carrier family 39 member 8 [Source:HGNC Symbol;Acc:HGNC:20862] | 6450 | -0.039 | -0.2476 | No |
| 50 | SLC4A8 | solute carrier family 4 member 8 [Source:HGNC Symbol;Acc:HGNC:11034] | 6455 | -0.039 | -0.2469 | No |
| 51 | SLC7A11 | solute carrier family 7 member 11 [Source:HGNC Symbol;Acc:HGNC:11059] | 6470 | -0.040 | -0.2470 | No |
| 52 | SLC25A1 | solute carrier family 25 member 1 [Source:HGNC Symbol;Acc:HGNC:10979] | 6509 | -0.043 | -0.2490 | No |
| 53 | SLC4A5 | solute carrier family 4 member 5 [Source:HGNC Symbol;Acc:HGNC:18168] | 6572 | -0.047 | -0.2526 | No |
| 54 | SLC25A23 | solute carrier family 25 member 23 [Source:HGNC Symbol;Acc:HGNC:19375] | 6676 | -0.054 | -0.2594 | No |
| 55 | SLC17A7 | solute carrier family 17 member 7 [Source:HGNC Symbol;Acc:HGNC:16704] | 6773 | -0.061 | -0.2654 | No |
| 56 | SLC6A1 | solute carrier family 6 member 1 [Source:HGNC Symbol;Acc:HGNC:11042] | 6970 | -0.078 | -0.2788 | No |
| 57 | SLC1A2 | solute carrier family 1 member 2 [Source:HGNC Symbol;Acc:HGNC:10940] | 7026 | -0.082 | -0.2811 | No |
| 58 | SLC22A6 | solute carrier family 22 member 6 [Source:HGNC Symbol;Acc:HGNC:10970] | 7030 | -0.082 | -0.2793 | No |
| 59 | SLC2A1 | solute carrier family 2 member 1 [Source:HGNC Symbol;Acc:HGNC:11005] | 7089 | -0.087 | -0.2816 | No |
| 60 | SLC52A1 | solute carrier family 52 member 1 [Source:HGNC Symbol;Acc:HGNC:30225] | 7145 | -0.092 | -0.2837 | No |
| 61 | SLC6A5 | solute carrier family 6 member 5 [Source:HGNC Symbol;Acc:HGNC:11051] | 7439 | -0.114 | -0.3038 | No |
| 62 | SLC6A13 | solute carrier family 6 member 13 [Source:HGNC Symbol;Acc:HGNC:11046] | 7463 | -0.115 | -0.3027 | No |
| 63 | ABCG2 | ATP binding cassette subfamily G member 2 (Junior blood group) [Source:HGNC Symbol;Acc:HGNC:74] | 7473 | -0.116 | -0.3005 | No |
| 64 | SLC26A1 | solute carrier family 26 member 1 [Source:HGNC Symbol;Acc:HGNC:10993] | 7618 | -0.128 | -0.3086 | No |
| 65 | SLC17A3 | solute carrier family 17 member 3 [Source:HGNC Symbol;Acc:HGNC:10931] | 7650 | -0.131 | -0.3078 | No |
| 66 | SLC36A1 | solute carrier family 36 member 1 [Source:HGNC Symbol;Acc:HGNC:18761] | 7670 | -0.132 | -0.3060 | No |
| 67 | ASIC3 | acid sensing ion channel subunit 3 [Source:HGNC Symbol;Acc:HGNC:101] | 7854 | -0.145 | -0.3167 | No |
| 68 | SLC25A5 | solute carrier family 25 member 5 [Source:HGNC Symbol;Acc:HGNC:10991] | 7877 | -0.146 | -0.3148 | No |
| 69 | CD36 | CD36 molecule [Source:HGNC Symbol;Acc:HGNC:1663] | 7985 | -0.154 | -0.3194 | No |
| 70 | OCA2 | OCA2 melanosomal transmembrane protein [Source:HGNC Symbol;Acc:HGNC:8101] | 8064 | -0.160 | -0.3215 | No |
| 71 | SLC17A6 | solute carrier family 17 member 6 [Source:HGNC Symbol;Acc:HGNC:16703] | 8119 | -0.164 | -0.3216 | No |
| 72 | SLC1A1 | solute carrier family 1 member 1 [Source:HGNC Symbol;Acc:HGNC:10939] | 8157 | -0.167 | -0.3204 | No |
| 73 | SLC13A2 | solute carrier family 13 member 2 [Source:HGNC Symbol;Acc:HGNC:10917] | 8259 | -0.176 | -0.3239 | No |
| 74 | SLC25A24 | solute carrier family 25 member 24 [Source:HGNC Symbol;Acc:HGNC:20662] | 8273 | -0.177 | -0.3205 | No |
| 75 | SLC35D1 | solute carrier family 35 member D1 [Source:HGNC Symbol;Acc:HGNC:20800] | 8408 | -0.186 | -0.3264 | No |
| 76 | SLC7A10 | solute carrier family 7 member 10 [Source:HGNC Symbol;Acc:HGNC:11058] | 8492 | -0.193 | -0.3281 | No |
| 77 | SLC1A7 | solute carrier family 1 member 7 [Source:HGNC Symbol;Acc:HGNC:10945] | 8504 | -0.194 | -0.3241 | No |
| 78 | SLC7A2 | solute carrier family 7 member 2 [Source:HGNC Symbol;Acc:HGNC:11060] | 8518 | -0.195 | -0.3203 | No |
| 79 | SLC25A10 | solute carrier family 25 member 10 [Source:HGNC Symbol;Acc:HGNC:10980] | 8552 | -0.198 | -0.3179 | No |
| 80 | SLC25A22 | solute carrier family 25 member 22 [Source:HGNC Symbol;Acc:HGNC:19954] | 8682 | -0.208 | -0.3229 | No |
| 81 | SLC6A9 | solute carrier family 6 member 9 [Source:HGNC Symbol;Acc:HGNC:11056] | 8704 | -0.209 | -0.3193 | No |
| 82 | SLC25A13 | solute carrier family 25 member 13 [Source:HGNC Symbol;Acc:HGNC:10983] | 8713 | -0.210 | -0.3147 | No |
| 83 | SLC35D2 | solute carrier family 35 member D2 [Source:HGNC Symbol;Acc:HGNC:20799] | 8714 | -0.210 | -0.3095 | No |
| 84 | SLC23A2 | solute carrier family 23 member 2 [Source:HGNC Symbol;Acc:HGNC:10973] | 8732 | -0.211 | -0.3056 | No |
| 85 | SLC44A4 | solute carrier family 44 member 4 [Source:HGNC Symbol;Acc:HGNC:13941] | 8853 | -0.221 | -0.3095 | No |
| 86 | ABCC5 | ATP binding cassette subfamily C member 5 [Source:HGNC Symbol;Acc:HGNC:56] | 9066 | -0.237 | -0.3202 | No |
| 87 | SLC25A32 | solute carrier family 25 member 32 [Source:HGNC Symbol;Acc:HGNC:29683] | 9170 | -0.246 | -0.3221 | No |
| 88 | SLC26A3 | solute carrier family 26 member 3 [Source:HGNC Symbol;Acc:HGNC:3018] | 9261 | -0.253 | -0.3229 | No |
| 89 | GJA1 | gap junction protein alpha 1 [Source:HGNC Symbol;Acc:HGNC:4274] | 9325 | -0.258 | -0.3214 | No |
| 90 | SLC26A2 | solute carrier family 26 member 2 [Source:HGNC Symbol;Acc:HGNC:10994] | 9523 | -0.276 | -0.3300 | No |
| 91 | SLC7A9 | solute carrier family 7 member 9 [Source:HGNC Symbol;Acc:HGNC:11067] | 9539 | -0.277 | -0.3242 | No |
| 92 | SLC1A3 | solute carrier family 1 member 3 [Source:HGNC Symbol;Acc:HGNC:10941] | 9656 | -0.288 | -0.3262 | No |
| 93 | SLC26A6 | solute carrier family 26 member 6 [Source:HGNC Symbol;Acc:HGNC:14472] | 9898 | -0.311 | -0.3373 | Yes |
| 94 | SLC25A38 | solute carrier family 25 member 38 [Source:HGNC Symbol;Acc:HGNC:26054] | 9904 | -0.312 | -0.3299 | Yes |
| 95 | SLC19A1 | solute carrier family 19 member 1 [Source:HGNC Symbol;Acc:HGNC:10937] | 9931 | -0.315 | -0.3241 | Yes |
| 96 | SLCO1B3 | solute carrier organic anion transporter family member 1B3 [Source:HGNC Symbol;Acc:HGNC:10961] | 10011 | -0.323 | -0.3223 | Yes |
| 97 | SLC13A3 | solute carrier family 13 member 3 [Source:HGNC Symbol;Acc:HGNC:14430] | 10096 | -0.330 | -0.3207 | Yes |
| 98 | SLC7A1 | solute carrier family 7 member 1 [Source:HGNC Symbol;Acc:HGNC:11057] | 10131 | -0.334 | -0.3150 | Yes |
| 99 | SLC6A7 | solute carrier family 6 member 7 [Source:HGNC Symbol;Acc:HGNC:11054] | 10185 | -0.339 | -0.3107 | Yes |
| 100 | SLC22A13 | solute carrier family 22 member 13 [Source:HGNC Symbol;Acc:HGNC:8494] | 10192 | -0.340 | -0.3027 | Yes |
| 101 | SLC7A8 | solute carrier family 7 member 8 [Source:HGNC Symbol;Acc:HGNC:11066] | 10228 | -0.344 | -0.2969 | Yes |
| 102 | SERINC5 | serine incorporator 5 [Source:HGNC Symbol;Acc:HGNC:18825] | 10352 | -0.356 | -0.2977 | Yes |
| 103 | SLC47A1 | solute carrier family 47 member 1 [Source:HGNC Symbol;Acc:HGNC:25588] | 10423 | -0.363 | -0.2941 | Yes |
| 104 | SLC38A3 | solute carrier family 38 member 3 [Source:HGNC Symbol;Acc:HGNC:18044] | 10443 | -0.366 | -0.2865 | Yes |
| 105 | BEST1 | bestrophin 1 [Source:HGNC Symbol;Acc:HGNC:12703] | 10469 | -0.370 | -0.2793 | Yes |
| 106 | SLCO2A1 | solute carrier organic anion transporter family member 2A1 [Source:HGNC Symbol;Acc:HGNC:10955] | 10546 | -0.378 | -0.2758 | Yes |
| 107 | SLC38A1 | solute carrier family 38 member 1 [Source:HGNC Symbol;Acc:HGNC:13447] | 10617 | -0.385 | -0.2717 | Yes |
| 108 | SLC1A6 | solute carrier family 1 member 6 [Source:HGNC Symbol;Acc:HGNC:10944] | 10626 | -0.386 | -0.2627 | Yes |
| 109 | SFXN1 | sideroflexin 1 [Source:HGNC Symbol;Acc:HGNC:16085] | 10880 | -0.414 | -0.2723 | Yes |
| 110 | CFTR | CF transmembrane conductance regulator [Source:HGNC Symbol;Acc:HGNC:1884] | 10920 | -0.420 | -0.2649 | Yes |
| 111 | SLC16A3 | solute carrier family 16 member 3 [Source:HGNC Symbol;Acc:HGNC:10924] | 10962 | -0.425 | -0.2575 | Yes |
| 112 | MFSD12 | major facilitator superfamily domain containing 12 [Source:HGNC Symbol;Acc:HGNC:28299] | 10965 | -0.426 | -0.2470 | Yes |
| 113 | SLC6A20 | solute carrier family 6 member 20 [Source:HGNC Symbol;Acc:HGNC:30927] | 10982 | -0.428 | -0.2376 | Yes |
| 114 | SERINC3 | serine incorporator 3 [Source:HGNC Symbol;Acc:HGNC:11699] | 11087 | -0.442 | -0.2348 | Yes |
| 115 | SLC22A14 | solute carrier family 22 member 14 [Source:HGNC Symbol;Acc:HGNC:8495] | 11139 | -0.448 | -0.2276 | Yes |
| 116 | SLC4A3 | solute carrier family 4 member 3 [Source:HGNC Symbol;Acc:HGNC:11029] | 11318 | -0.474 | -0.2298 | Yes |
| 117 | SLC6A8 | solute carrier family 6 member 8 [Source:HGNC Symbol;Acc:HGNC:11055] | 11453 | -0.496 | -0.2279 | Yes |
| 118 | ABCC1 | ATP binding cassette subfamily C member 1 [Source:HGNC Symbol;Acc:HGNC:51] | 11462 | -0.497 | -0.2162 | Yes |
| 119 | SLCO3A1 | solute carrier organic anion transporter family member 3A1 [Source:HGNC Symbol;Acc:HGNC:10952] | 11564 | -0.510 | -0.2114 | Yes |
| 120 | SLC4A7 | solute carrier family 4 member 7 [Source:HGNC Symbol;Acc:HGNC:11033] | 11587 | -0.513 | -0.2004 | Yes |
| 121 | SLC17A9 | solute carrier family 17 member 9 [Source:HGNC Symbol;Acc:HGNC:16192] | 11596 | -0.515 | -0.1882 | Yes |
| 122 | SLC25A17 | solute carrier family 25 member 17 [Source:HGNC Symbol;Acc:HGNC:10987] | 11669 | -0.527 | -0.1807 | Yes |
| 123 | SLC22A7 | solute carrier family 22 member 7 [Source:HGNC Symbol;Acc:HGNC:10971] | 11678 | -0.528 | -0.1682 | Yes |
| 124 | SLC38A2 | solute carrier family 38 member 2 [Source:HGNC Symbol;Acc:HGNC:13448] | 11722 | -0.536 | -0.1582 | Yes |
| 125 | SLC5A6 | solute carrier family 5 member 6 [Source:HGNC Symbol;Acc:HGNC:11041] | 11809 | -0.551 | -0.1512 | Yes |
| 126 | SLC22A11 | solute carrier family 22 member 11 [Source:HGNC Symbol;Acc:HGNC:18120] | 11826 | -0.554 | -0.1387 | Yes |
| 127 | SLC6A11 | solute carrier family 6 member 11 [Source:HGNC Symbol;Acc:HGNC:11044] | 11916 | -0.575 | -0.1313 | Yes |
| 128 | SLC6A6 | solute carrier family 6 member 6 [Source:HGNC Symbol;Acc:HGNC:11052] | 12128 | -0.626 | -0.1323 | Yes |
| 129 | SLC7A5 | solute carrier family 7 member 5 [Source:HGNC Symbol;Acc:HGNC:11063] | 12203 | -0.646 | -0.1220 | Yes |
| 130 | SLC1A5 | solute carrier family 1 member 5 [Source:HGNC Symbol;Acc:HGNC:10943] | 12501 | -0.748 | -0.1267 | Yes |
| 131 | SLC16A7 | solute carrier family 16 member 7 [Source:HGNC Symbol;Acc:HGNC:10928] | 12545 | -0.771 | -0.1109 | Yes |
| 132 | SLC3A2 | solute carrier family 3 member 2 [Source:HGNC Symbol;Acc:HGNC:11026] | 12587 | -0.789 | -0.0944 | Yes |
| 133 | SLCO4A1 | solute carrier organic anion transporter family member 4A1 [Source:HGNC Symbol;Acc:HGNC:10953] | 12593 | -0.792 | -0.0751 | Yes |
| 134 | SLC19A2 | solute carrier family 19 member 2 [Source:HGNC Symbol;Acc:HGNC:10938] | 12600 | -0.798 | -0.0557 | Yes |
| 135 | SLC2A3 | solute carrier family 2 member 3 [Source:HGNC Symbol;Acc:HGNC:11007] | 12604 | -0.800 | -0.0360 | Yes |
| 136 | SLC52A2 | solute carrier family 52 member 2 [Source:HGNC Symbol;Acc:HGNC:30224] | 12735 | -0.899 | -0.0239 | Yes |
| 137 | SLC26A10 | solute carrier family 26 member 10 [Source:HGNC Symbol;Acc:HGNC:14470] | 12899 | -1.475 | 0.0001 | Yes |
Table: GSEA details [plain text format]

  

Fig 2: GOMF\_ORGANIC\_ANION\_TRANSMEMBRANE\_TRANSPORTER\_ACTIVITY      
 Blue-Pink O' Gram in the Space of the Analyzed GeneSet

  

Fig 3: GOMF\_ORGANIC\_ANION\_TRANSMEMBRANE\_TRANSPORTER\_ACTIVITY: Random ES distribution      
 Gene set null distribution of ES for **GOMF\_ORGANIC\_ANION\_TRANSMEMBRANE\_TRANSPORTER\_ACTIVITY**
